# Successful treatment of nonunion with an Ilizarov ring fixator after ankle fracture for Charcot arthropathy: a case report

**DOI:** 10.1186/1756-0500-7-503

**Published:** 2014-08-07

**Authors:** Koji Nozaka, Yoichi Shimada, Yoshiaki Kimura, Shin Yamada, Takeshi Kashiwagura, Tsutomu Sakuraba, Ikuko Wakabayashi

**Affiliations:** 1 Department of Orthopedic Surgery, Akita University Graduate School of Medicine, 1-1-1 Hondo, Akita 010-8543, Japan; 2 Department of Orthopedic Surgery, Akita City Hospital, 4-30 kawamoto matsuokacho, Akita 010-0933, Japan

**Keywords:** Ilizarov ring fixator, Ankle fracture, Charcot arthropathy, Nonunion, Diabetes

## Abstract

**Background:**

Ankle fractures in patients with diabetes mellitus have long been recognized as a challenge to orthopedic surgeons. Nonunion and lengthy wound healing in high-risk patients with diabetes, particularly patients with peripheral arterial disease and renal failure, occur secondary to several clinical conditions and are often fraught with complications. Whether diabetic ankle fractures are best treated noninvasively or surgically is controversial.

**Case presentation:**

A 53-year-old Japanese man fractured his right ankle. The fractured ankle was treated nonsurgically with a plaster cast. Although he remained non-weight-bearing for 3 months, radiography at 3 months showed nonunion. The nonunion was treated by Ilizarov external fixation of the ankle. The external fixator was removed 99 days postoperatively, at which time the patient exhibited anatomical and functional recovery and was able to walk without severe complications.

**Conclusion:**

In patients with diabetes mellitus, severe nonunion of ankle fractures with Charcot arthropathy in which the fracture fragment diameter is very small and the use of internal fixation is difficult is a clinical challenge. Ilizarov external fixation allows suitable fixation to be achieved using multiple Ilizarov wires.

## Background

Increasing numbers of patients are being diagnosed with diabetes mellitus, and they are living longer because of improvements in treatment. Ankle fractures in patients with diabetes mellitus have long been recognized as a challenge to orthopedic surgeons [1]. Nonunion and lengthy wound healing in high-risk patients with diabetes [2], particularly patients with peripheral arterial disease and renal failure, occur secondary to several clinical conditions and are often fraught with complications [3].

## Case presentation

**Figure 1:**
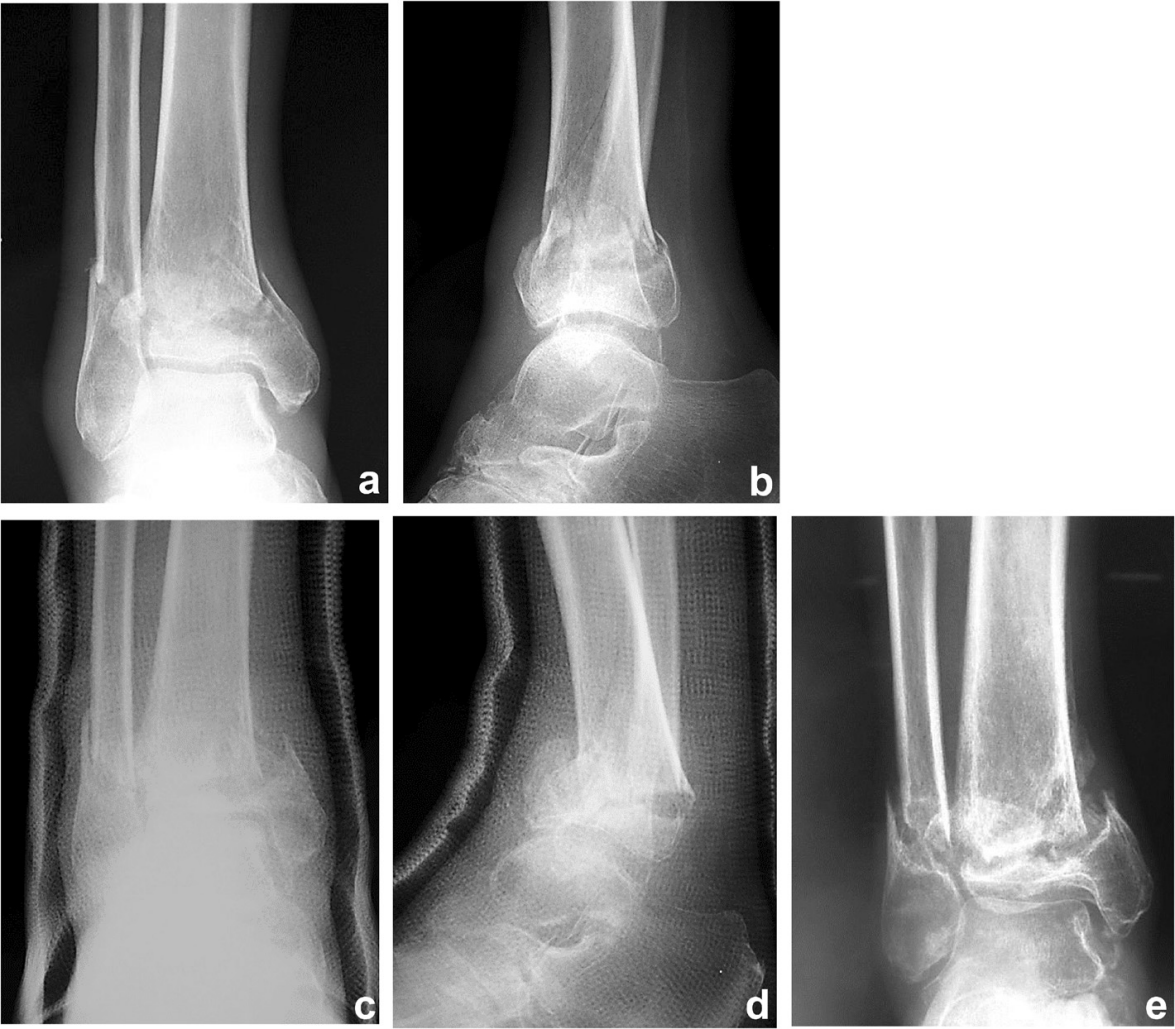
**Preoperative radiographs. (a, b)** The initial plain X-ray showed a right ankle fracture (AO type 43-A1.3). **(c, d)** Radiographs 1.5 months after the injury. **(e)** Radiograph of nonunion 3 months after injury on initial consultation.

**Figure 2:**
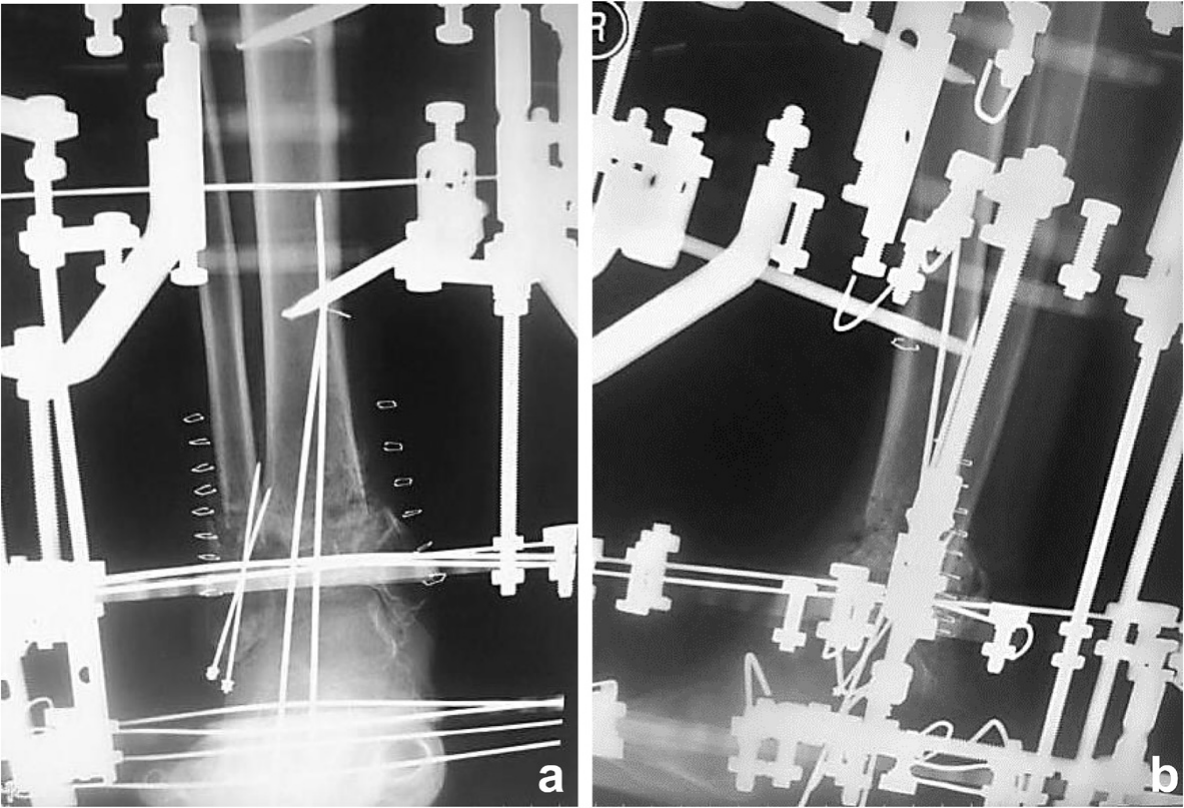
**Postoperative radiographs. (a, b)** Anteroposterior- and lateral-view plain radiographs after surgery, showing anatomical reduction.

**Figure 3:**
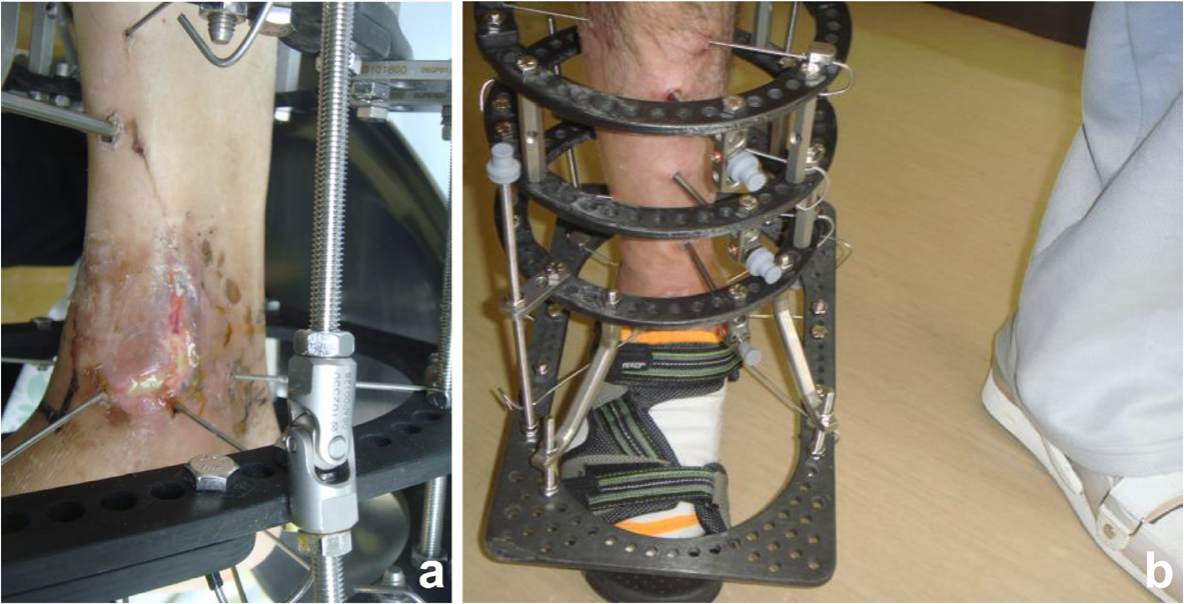
**Postoperative appearance. (a)** Postoperative skin necrosis at the medial skin incision. **(b)** The external fixator of the foot was removed 6 weeks after surgery.

**Figure 4:**
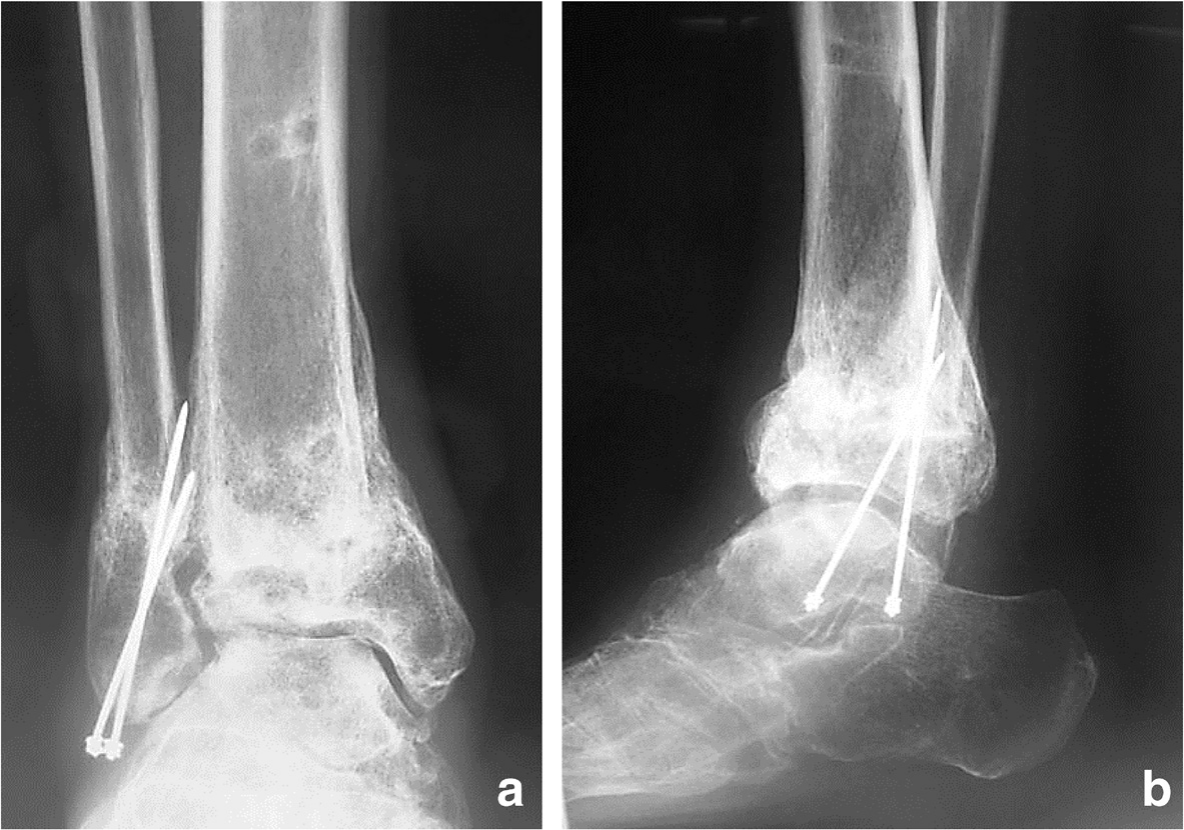
**Postoperative radiographs. (a, b)** Radiograph after removal of the external fixator, showing bone union.

A 53-year-old Japanese man injured his right ankle while walking on a wet road. At the time of the injury, he had been walking with a T-cane following surgical repair of a left hip fracture and had Charcot knee arthropathy in his left knee. He felt pain in the right ankle for 1 week after the injury and presented to another hospital for evaluation. The initial plain X-ray showed a right ankle fracture (Arbeitsgemeinschaft für Osteosynthesefragen/Orthopaedic Trauma Association [AO/OTA] type 43-A1.3) (Figure 1a, b). The patient also had severe diabetes mellitus, anemia, and chronic kidney disease. He had stopped self-injection of insulin and developed severe hyperglycemia. On physical examination, he had extremely weak pulses and many small ulcers on his lower limbs. Laboratory testing revealed a high level of hemoglobin A1c (10.9%; reference value, <5.8%), low level of hemoglobin (8.9 g/dL; reference range, 13.5–17.0 mg/dL), and high level of serum creatinine (3.8 mg/dL; reference range, 0.8–1.3 mg/dL) (Table 1). The bone mineral density of the lumbar spine (L2–4) (0.549 g/cm^2^; T-score, -3.78 S.D.) and proximal femur (0.622 g/cm^2^; T-score, -2.57S.D.) confirmed a diagnosis of osteoporosis. The nerve conduction velocity was very slow, as seen in patients with diabetes mellitus with generalized peripheral neuropathy. Another doctor considered that his anesthetic risk was high; therefore, he was treated nonsurgically. His ankle was placed in a total cast, and he was advised to avoid bearing weight on his right leg. His ankle developed slight swelling and redness approximately 2 weeks after casting; at 1.5 months, his ankle was swollen and erythematous with minor discomfort (Figure 1c, d). Another doctor continued nonsurgical treatment with the plaster cast. Although the patient continued to walk without weight-bearing for 3 months, radiologic assessment at 3 months showed no signs of healing (Figure 1e). The treating doctor considered that the distal tibia fracture fragment was too small to fix with internal fixation, and the patient’s soft tissue condition was poor. The patient was then transferred to our department. We decided to proceed with osteosynthesis with an Ilizarov ring fixator to preserve the joint function. Given the patient’s condition, the risk of skin disorders with the use of bulky internal fixation materials appeared to be high. After admission to our institute, Ilizarov ring fixator surgery was performed with the patient under general anesthesia in the supine position. Five rings were used for the Ilizarov fixator. Maintenance of axial alignment and rotation and correct length adjustment were checked using intensification. The foot was fixed to a foot ring connected to the tibial external fixator (Figure 2a, b). Both ends of the bone fragments were then chipped into small pieces at the injured site using a hammer and osteotome without peeling off the periosteum [4]. After chipping, the sites of nonunion in the fibula and tibia were shortened until they were no longer recognizable. A 1.8-mm Ilizarov wire was passed parallel to the articular surface on the anteroposterior X-ray view of the tibial epiphysis. Five wires were inserted onto the distal tibial ring. The wires were fixed to the rings of the fixator and tensioned. The foot was fixed in the neutral position to avoid both supination and equinus. Postoperative skin necrosis on the medial skin incision was successfully treated with antibiotic ointment (Figure 3a). Full weight-bearing walking was permitted 14 days postoperatively. The external fixator of the foot was removed 6 weeks after surgery (Figure 3b). Radiographs showed healing of the fracture 99 days postoperatively (Figure 4a, b). At the 2-year postoperative follow-up, the patient was satisfied with the outcome and was able to walk with a T-cane. Clinical outcomes were measured using the postoperative American Orthopedic Foot & Ankle Society scale ankle/hindfoot scale score (postoperative score of 94), Short Form-36 (postoperative physical component summary subscore of 47.7, mental component summary subscore of 59.3), and visual analog scale for pain (postoperative score of 0). At the last visit, the patient exhibited 0° and 30° dorsal and plantar flexion, respectively, and the range of motion of the operated ankle almost matched that of the unoperated ankle.

**Table 1:** Patient’s pretreatment laboratory findings

WBC	5800	/μL	CRP	0.98	mg/dl
RBC	280 × 10^4^	/μL	LDH	238	U/L
Hb	8.9	g/dL	UA	6.8	mg/dL
Plt	12.7 × 10^4^	/μL	BUN	37.6	mg/dL
PT	11.9	sec	Cr	3.8	mg/dL
AST	32	IU/L	Na	141	mEq/l
ALT	36	IU/L	K	3.8	mEq/l
TP	5.7	g/dL	Ca	10.9	mg/dL
Alb	2.7	g/dL	P	5.8	mg/dL
TBIL	0.5	mg/dL	BS	385	mg/dl
ALP	248	IU/L	TG	1476	mg/dl
Γ-GTP	20	IU/L	HbA1c	10.9	mg/dl

WBC, white blood cells; RBC, red blood cells; Hb, hemoglobin; Plt, platelets; PT, prothrombin time; AST, aspartate aminotransferase; ALT, alanine aminotransferase; TP, total protein; Alb, albumin; TBIL, total bilirubin; ALP, alkaline phosphatase; γ-GTP, gamma glutamyl transpeptidase; CRP, C-reactive protein; LDH, lactate dehydrogenase; UA, uric acid; BUN, blood urea nitrogen; Cr, creatinine; Na, sodium; K, potassium; Ca, calcium; P, phosphorus; BS, blood sugar; TG, triglycerides; HbA1c, hemoglobin A1c.

## Conclusions

Increasing numbers of patients are being diagnosed with diabetes, and they are living longer because of improvements in treatment [1-3]. Ankle fractures in patients with diabetes mellitus have long been recognized as a challenge to orthopedic surgeons. Nonunion and lengthy wound healing in high-risk patients with diabetes, particularly in those with peripheral arterial disease and renal failure, as in the present case, are often fraught with complications [1-3]. Whether diabetic ankle fractures are best treated noninvasively or surgically is controversial [2]. Some previous studies have shown that nondisplaced fractures in high-risk patients can be managed nonsurgically in a cast [5,6]. Treatment entails casting with non-weight-bearing restriction until fracture healing is demonstrated.

McCormack *et al*. described 26 ankle fractures in patients with diabetes; 19 were treated surgically, and seven were immobilized in casts. The surgical group included one wound complication (5%), four infections (21%) leading to two amputations (11%), and two deaths (11%), for an overall complication rate of 47% [6]. Because the present patient was a high-risk patient with diabetes mellitus, he was managed nonsurgically with a cast in another hospital. Rigid internal fixation was considered to have been difficult because the diameter of the distal fragment was very small. Furthermore, bone softening of the small distal fragment was noted at surgery. Ilizarov external fixation for severe ankle fracture in patients with diabetes mellitus in which the diameter of the fracture fragment is very small and the use of internal fixation is difficult allows suitable fixation to be achieved using multiple Ilizarov wires. It was thought that the frame should extend to the foot (ankle-joint bridging) so that weight-bearing forces would not be transmitted proximally by the talus. This protects the articular surface and decreases problems associated with loosening and infection of the distal tibial pins. It also increases the safety of earlier weight-bearing. After sufficient fracture healing had occurred, the foot ring was removed to allow ankle motion and progressive weight-bearing. More progenitor cells from bone marrow were probably introduced at the site of nonunion by pulverizing the complete bone; more cytokines, such as bone morphogenic protein or basic fibroblast growth factor, were likely introduced from the bone matrix as well [4]. Furthermore, we anticipated that the increased skin tension associated with the use of a locking plate could be prevented. Ankle fractures in patients with diabetes mellitus are characterized by thinning of the subcutaneous tissue, poor dermal extensibility, and senile skin atrophy [7]. The skin also becomes more fragile and susceptible to trauma in these patients, leading to more lacerations and bruising [8].

Lower-profile metallic implants have significantly reduced soft tissue complications in recent years; however, cases of implant-related soft tissue problems are still encountered, particularly in patients with Charcot arthropathy [9]. Some techniques address osteopenia or wound necrosis, which are important problems in diabetic patients with ankle fractures. In the present case, the patient only underwent daily showering of the pin tract of the external fixator; no other physical pin cleaning was performed [10], and no pin tract infection developed. Lovisetti *et al*. reported no cases of pseudoarthrosis or deep infection [11-13]. They attributed their 100% union rate to meticulous preservation of soft tissues in the fracture zone. Treatment of ankle fractures in patients with diabetes mellitus by circular external fixation allows for less soft tissue dissection and is a reliable method for achieving stabilization and healing of distal tibial fractures with fewer soft tissue complications. Use of the Ilizarov external fixator is a safe method for ankle fractures in patients with diabetes mellitus and has the advantage of rigid fixation.

Successful treatment of nonunion despite the presence of Charcot ankle arthropathy might be attributed to chipping. It is believed that immediate weight-bearing improves limb circulation and enhances the healing process based on the fact that the speed of fracture healing is usually proportional to the amount of available circulation to and between the fragments [14]. The present case provides satisfactory evidence that this procedure can be used to successfully manage such nonunions after ankle fracture with Charcot arthropathy. In conclusion, Ilizarov external fixation for severe nonunion of ankle fractures in patients with diabetes mellitus in which the diameter of the fracture fragment is very small and the use of internal fixation is difficult allows suitable fixation to be achieved using multiple Ilizarov wires.

## Consent

Written informed consent was obtained from the patient for publication of this Case Report and any accompanying images. A copy of the written consent is available for review by the Editor-in-Chief of this journal.

## Competing interests

The authors declare that they have no competing interests. There is no substantial direct or indirect commercial financial incentive associated with publishing this article.

## Authors’ contributions

KN performed the surgery. YS assisted with the surgery and helped to draft the manuscript. SY and IW helped to draft the manuscript. YK, TK, and TS assisted with the surgery. All authors read and approved the final manuscript.
